# PEP-1-MsrA ameliorates inflammation and reduces atherosclerosis in apolipoprotein E deficient mice

**DOI:** 10.1186/s12967-015-0677-8

**Published:** 2015-09-26

**Authors:** Yao Wu, Guanghui Xie, Yanyong Xu, Li Ma, Chuanfeng Tong, Daping Fan, Fen Du, Hong Yu

**Affiliations:** Department of Biochemistry and Molecular Biology, Hubei Provincial Key Laboratory of Developmentally Originated Disease, Wuhan University School of Basic Medical Sciences, 185 Donghu Road, Bldg. 2, 2-209, Wuhan, 430071 Hubei China; Cardiology Division of Wuhan University Zhongnan Hospital, Wuhan, China; Department of Cell Biology and Anatomy, School of Medicine, University of South Carolina, Columbia, SC USA

**Keywords:** Methionine sulfoxide reductase A, Atherosclerosis, PEP-1, ROS, Inflammation, Macrophages, Penetrating fusion protein

## Abstract

**Background:**

Methionine sulfoxide reductase A (MsrA) is a potent intracellular oxidoreductase and serves as an essential factor that protects cells against oxidative damage. However, therapeutic use of exogenous MsrA in oxidative stress-induced diseases is limited, because it cannot enter the cells. The aim of this study is to investigate whether MsrA with PEP-1, a cell penetrating peptide, fused to its N-terminus can protect against oxidative stress in macrophages and can attenuate atherosclerosis in apolipoprotein E deficient (apoE^−/−^) mice.

**Methods:**

MsrA and the fusion protein PEP-1-MsrA were expressed and purified using a pET28a expression system. Transduction of the fusion protein into macrophages was confirmed by Western blot and immunofluorescence staining. Intracellular reactive oxygen species (ROS) and apoptosis levels were measured by flow cytometry. In in vivo study, MsrA or PEP-1-MsrA proteins were intraperitoneally injected into apoE^−/−^ mice fed a Western diet for 12 weeks. Plasma lipids levels, inflammatory gene expression, and paraoxonase-1 (PON1) and superoxide dismutase (SOD) activities were assessed. Atherosclerotic lesions were analyzed by Oil Red O staining and immunohistochemistry.

**Results:**

PEP-1-MsrA could penetrate the cells and significantly reduced intracellular ROS levels and apoptosis in H_2_O_2_-treated macrophages. It also decreased TNFα and IL-1β mRNA levels and increased the IL-10 mRNA level in lipopolysaccharide-treated macrophages. In in vivo study, PEP-1-MsrA injection significantly increased plasma PON1 and SOD activities and decreased plasma monocyte chemoattractant protein 1 (MCP-1) level compared to the injection of vehicle control or MsrA. In PEP-1-MsrA injected mice, hepatic PON1 levels were increased, while the expression of TNFα and IL-6 mRNA in the liver was suppressed. Although plasma total cholesterol and triglyceride levels did not change, the aortic atherosclerosis in PEP-1-MsrA treated mice was significantly reduced. This was accompanied by a reduction of total and apoptotic macrophages in the lesions.

**Conclusion:**

Our study provides evidence that PEP-1-MsrA may be a potential therapeutic agent for atherosclerosis-related cardiovascular diseases.

**Electronic supplementary material:**

The online version of this article (doi:10.1186/s12967-015-0677-8) contains supplementary material, which is available to authorized users.

## Background

Cardiovascular disease (CVD), primarily induced by atherosclerosis, is the most common cause of morbidity and disability worldwide. Atherosclerosis is now considered a chronic inflammatory disease associated with oxidative stress and dyslipidemia [1–3]. Numerous studies have documented that among the multifactorial causes of atherosclerosis, reactive oxygen species (ROS) play important roles in its pathogenesis by stimulating the oxidation and modification of lipids and proteins, which can lead to foam cell formation and atherosclerotic plaque progression in the blood vessel wall [4].

ROS, including superoxide anion, hydroxyl radical and hydrogen peroxide (H_2_O_2_), are constantly generated by cells as byproducts of normal metabolism and highly localized within the cells [5, 6]. Cells contain a series of antioxidant defenses such as superoxide dismutase (SOD), catalase (CAT) and glutathione peroxidase that directly detoxify ROS and reduce their effects. The imbalance between ROS generation and the antioxidant system in vascular cells occurs due to a change in the overall redox balance and in the modification of target molecules, resulting in inflammation and atherogenesis. There is increasing evidence that a therapeutic approach targeting antioxidant enzymes could decrease the progression of atherosclerosis [7].

Sulfur-containing methionine (Met), either free or in proteins, is an essential amino acid and a sensitive target for oxidants. Met is easily oxidized into methionine-*S*-sulfoxide (MetSO) which can be reduced back to Met by a ubiquitous intracellular enzyme named methionine sulfoxide reductase A (MsrA). In turn, oxidized MsrA can be reduced by the cellular thioredoxin (Trx), thioredoxin reductase and NADPH system [8]. Recent studies have demonstrated that MsrA is involved in oxidative stress and age associated diseases [9], and enhanced MsrA might be an important approach for the prevention of many oxidative stress-induced diseases, such as hypoxia/reoxygenation and ischemia/reperfusion injury [10, 11], cataracts [12], diabetes mellitus [13], Alzheimer’s disease [14, 15] and atherosclerosis related coronary artery disease [16, 17].

MsrA plays protective roles by maintaining intracellular redox homeostasis; however, therapeutic use of exogenous MsrA is limited, because this enzyme is poorly taken up by cells [18]. Cell penetrating peptides (CPPs) or protein transduction domains (PTDs) were designed to deliver large proteins into mammalian cells [19]. A 21-amino acid peptide, PEP-1, is a new type of CPP [20], and has been reported to be able to successfully transfer a wide range of therapeutic proteins such as SOD [21], CAT [22], paraoxonase-1 (PON1) [23] and peroxiredoxin2 [24] into cells and thus to achieve their intracellular biological activities [25]. Although the precise mechanism is as yet unclear, PEP-1 fusion proteins have been used in research to protect against many diseases including neuronal disease, myocardial ischemic/reperfusion injury and skin inflammation [26–28].

The goal of this study is to investigate whether the fusion protein PEP-1-MsrA can efficiently enter cells to protect against oxidative stress and to attenuate atherosclerosis in apolipoprotein E deficient (apoE^−/−^) mice, providing evidence that PEP-1-MsrA may be a potential therapeutic agent for atherosclerosis-related cardiovascular diseases.

## Methods

### Expression and purification of MsrA and PEP-1-MsrA fusion protein in *E. coli*

The plasmid containing the human MsrA cDNA sequence without a signal peptide was provided by Prof. Stefan H. Heinemann (Jena Friedrich Schiller University, Germany) and the pET15b/PEP-1 plasmid was provided by Prof. Jia-Ning Wang (Hubei University of Medicine, China). The pET28a vectors were purchased from Novagen (Merck Millipore, Germany). To generate the recombinant pET28a/PEP-1-MsrA vector, a 642-bp cDNA fragment of human MsrA, digested at *Bam*HI and *Hind*III sites, from pQE30/MsrA was inserted into a pET15b/PEP-1 plasmid. Then the PEP-1-MsrA fragment was digested at *Nde*I and *Hind*III sites and inserted into a pET28a vector. A pET28a/MsrA vector was also constructed as control. The recombinant vectors were verified by DNA sequencing.

To produce PEP-1-MsrA or MsrA proteins, plasmids were transformed into *E. coli* BL21 (DE3). The transformed cells were grown in LB medium containing 10 µg/ml kanamycin at 37 °C to an A_600_ value of 0.6–0.8 and induced with 1 mM isopropyl β-d-1-thiogalactoside (IPTG) at 30 °C for 4–6 h. Then the bacteria were harvested and lysed by a low temperature ultra-high pressure continuous flow cell disrupter (JNBIO, China) in lysis buffer (pH 8.0, 50 mM NaH_2_PO_4_, 400 mM NaCl, 10 mM imidazole including with 3 μg/ml lysozyme, 1 μg/ml DNase I and 5 μM PMSF). The proteins were purified by affinity chromatography using Ni-NTA Agarose (Qiagen, Germany) according to the manufacturer’s instructions. The purity was analyzed by 10 % SDS-PAGE with Coomassie Blue Staining. Concentrations of the purified proteins were determined by the Lowry method using a *DC* protein assay kit (Bio-Rad, USA).

### Circular dichroism measurements

Circular dichroism (CD) spectra of proteins were determined using a Jasco J-810 spectropolarimeter (Jasco Corp., Japan) with a 1 mm light-path quartz cell. The far-UV CD spectra were recorded from 190 to 250 nm. PEP-1-MsrA or MsrA protein solutions were prepared at a final concentration of 15 μM in 100 mM phosphate buffer (PB, pH 7.5). The averaged spectra of several scans were corrected relative to the buffer blank. Measurements were made at room temperature.

### MsrA activity assay

MsrA activity was measured using methyl sulfoxides (DMSO, Sigma-Aldrich, USA) as a substrate as described by Wu [29]. The reaction system (pH 8.0) contained 500 μM DMSO, 10 mM MgCl_2_, 30 mM KCl and 50 μM DTT in 25 mM Tris–HCl to which 3.6 μM of the heat-inactivated or active MsrA or PEP-1-MsrA was added. To terminate the reaction, 100 μl of reaction mixture and 100 μl of 4 M 5,5′-dithiobis (DTNB, Sigma-Aldrich, USA) were added and the mixture was incubated for 10 min at 37 °C. The A_412_ value was then recorded at 0 and 10 min after the end of the incubation period. The decrease in A_412_ value was calculated (ΔA = ΔA_0min_ − ΔA_10min_). The heat-inactivated MsrA (control) was defined as ΔA_Control_, while active MsrA was named ΔA_Total_. MsrA activity was calculated using the formula: ΔA_MsrA_ = ΔA_Total_ − ΔA_Control_. PEP-1-MsrA activity was normalized with MsrA.

### Cell culture

Murine macrophage cell line Raw 264.7 cells and human HeLa cells were obtained from the Animal Biosafety Level 3 Laboratory (ABSL-III) at Wuhan University, China. Mouse peritoneal macrophages were harvested by peritoneal lavage 3–4 days after intraperitoneal injection of 3 ml 3 % thioglycollate [30]. The cells were cultured in Dulbecco’s modified Eagle medium (DMEM, ThermoFisher, USA) containing 10 % fetal bovine serum (FBS, Gibco, USA) and antibiotics (100 mg/ml stre*p*tomycin, 100 U/ml penicillin, Beyotime, China) at 37 °C. Cells were seeded in plates and incubated with serum-free DMEM at 37 °C overnight before further treatment.

### Transduction of PEP-1-MsrA protein into cells

MTT method was performed to test the cytotoxicity of the proteins. HeLa cells were treated with MsrA or PEP-1-MsrA (0–18 μM) for 72 h. To determine the transduction efficiency of PEP-1-MsrA, peritoneal macrophages were treated with various concentrations of PEP-1-MsrA or MsrA protein (0.5–8 μM) for 1 h. Then cells were washed with PBS and harvested for Western blot analysis. The cells were also treated with 1 μM of proteins for various times (5 min–24 h) to examine the stability of PEP-1-MsrA by Western blot analysis.

We further detected the intracellular distribution of transduced protein using an immunofluorescence assay. Briefly, peritoneal macrophages were seeded on coverslips and treated with selected concentrations of PEP-1-MsrA or MsrA protein (1–4 μM) for 1 h, and then washed with PBS at least three times and fixed with 4 % paraformaldehyde for 10 min at 37 °C. The cells were incubated with the primary antibody (anti-MsrA antibody, Abcam, USA) overnight at 4 °C, then with FITC-conjugated goat anti-rabbit IgG (1:200) for 2 h at room temperature in the dark. Nuclei were stained with 300 nM DAPI (Sigma-Aldrich, USA) for 15 min at 37 °C. The intracellular localization of MsrA protein was analyzed with confocal microscopy (Olympus FluoView FV1000, Japan).

### Determination of intracellular ROS levels

Intracellular ROS levels were detected by the sensitive fluorescent dye 2′,7′-dichlorofluorescin diacetate (DCFH-DA, Sigma-Aldrich, USA) as described in previous studies [23, 28]. RAW264.7 cells were pre-incubated with PEP-1-MsrA or MsrA protein (1 µM) for 1 h, and then exposed to 1 mM H_2_O_2_ for 1 h. After washing with PBS, cells were incubated with 20 µM DCFH-DA for 30 min at 37 °C. The DCF fluorescence intensity was measured by flow cytometry (FACS Aria™ III system, BD, USA).

### Cell death assays

Cell death was detected by Annexin V and propidium iodide (PI) binding assay. Peritoneal primary macrophages were pre-treated with PEP-1-MsrA and MsrA protein (1 µM) for 1 h before incubated with 1 mM H_2_O_2_ for 1 h. Cells were resuspended in Annexin-V binding buffer and incubated with Annexin V-FITC and PI for 15 min at room temperature, according to the procedure for the Annexin-V-fluorescein (Annexin-V-FITC) Apoptosis Detection Kit (Bestbio, China). Total cell death was analyzed by flow cytometry.

### Western blot analysis

Harvested cells or frozen tissues were lysed by RIPA (Beyotime Institute of Biotechnology, China) with 1 % proteinase inhibitors (Roche, Germany) for Western blot analysis. Appropriate amounts of proteins were loaded and separated by 10 % SDS-PAGE and transferred onto a nitrocellulose membrane. Protein expression was detected using primary antibodies, anti-MsrA antibody or anti-PON1 antibody (Abcam, USA) (1:1000), followed by horseradish peroxidase (HRP)-conjugated secondary antibody (Santa Cruz Biotechnology, USA) (1:10,000). Signal was detected using an enhanced chemiluminescence kit (ECL, GE Healthcare, USA). The band densitometry was analyzed with Image J software (NIH, USA).

### Quantitative real-time PCR (qPCR)

Mouse peritoneal macrophages were co-cultured with 25 ng/ml LPS and 1 µM PEP-1-MsrA or MsrA proteins for 3 h, and then harvested with Trizol reagent (Invitrogen, USA). Mouse liver tissue was also homogenized with Trizol reagent. Total RNA was extracted using an RNeasy kit (Qiagen, Germany) and reverse transcribed into cDNA using a PrimerScript^®^ RT reagent Kit with gDNA Eraser (TaKaRa, Japan). Target mRNA levels were measured by qPCR with the CFX96 Touch™ Real-Time PCR Detection System (Bio-Rad, USA) according to the manufacturer’s instructions. Mouse genes were normalized with 18s RNA as an endogenous control. Primers used were as follows: mouse 18s RNA: 5′ CGCGGTTCTATTTTGTTGGT 3′ (forward) and 5′ AGTCGGCATCGTTTATGGTC 3′ (reverse); mouse IL-1β: 5′ GCCCATCCTCTGTGACTCAT 3′ (forward) and 5′ AGGCCACAGGTATTTTGTCG 3′ (reverse); mouse TNFα: 5′ CGTCAGCCGATTTGCTATCT 3′ (forward) and 5′ CGGACTCCGCAAAGTCTAAG 3′ (reverse); mouse IL-10: 5′ AGCCTTATCGGAAATGATCCAGT 3′ (forward) and 5′ GGCCTTGTAGACACCTTGGT 3′ (reverse); mouse PON1: 5′ TGGTGGTAAACCATCCAGACTC 3′ (forward) and 5′ TGTGATGGTTTTCAGATGCAAG 3′ (reverse); and mouse IL-6: 5′ AGTTGCCTTCTTGGGACTGA 3′ (forward) and 5′ TCCACGATTTCCCAGAGAAC 3′ (reverse).

### Mice

ApoE^−/−^ mice on a C57BL/6 background were purchased from Vital River Laboratory Animal Technology Company, China, and housed in microisolator cages in the Wuhan University Animal Center. Mice were fed in a temperature-controlled facility (temperature 22 ± 1 °C, humidity 60 %, 12 12-h dark-light cycle) with free access to food and water. Animal care and experimental procedures were performed under the regulations of the Institutional Animal Care and the Ethics Committee for Animal Experiments of Wuhan University, in accordance with the Guidelines for the Care and Use of Laboratory Animals of the Chinese Animal Welfare Committee. Thirty male apoE^−/−^ mice at 21 weeks of age were randomly divided into three groups and intraperitoneally injected with one of the two target proteins (dose of 5.5 nmol per mouse every 36 h) or 10 mM phosphate buffered saline (PBS, pH 7.4), respectively. Mice were fed with AIN76A Western diet (HFK bioscience company, China) for 12 weeks to accelerate the development of atherosclerosis.

### Determination of biochemical parameters in plasma and liver

Blood samples were collected from mice after overnight fasting by retro-orbital venous plexus puncture at 4 week intervals. Plasma was immediately separated by centrifugation at 10,000×*g* for 10 min at 4 °C. Total cholesterol (TC) and triglyceride (TG) levels in fresh plasma were measured by enzymatic colorimetric assay kits (Mind Bioengineering, China). The remaining plasma was stored at −80 °C for other analysis. Plasma MCP-1 levels were determined by enzyme-linked immunosorbent assay (ELISA) kits (eBioscience, USA) according to the manufacturer’s instructions. Plasma PON1 activity was measured using paraoxon (Sigma-Aldrich, USA) as a substrate as described previously [31]. Plasma SOD activity was measured using SOD Detection Kit (Nanjing Jiancheng Bioengineering Institute, China). Frozen mouse liver samples were lysed by RIPA with 1 % proteinase inhibitors for Western blot analysis. Liver homogenates treated with Trizol reagent were used for qPCR analysis of target mRNA levels.

### Histochemical and immunocytochemical analyses of atherosclerotic lesions

Mice were intraperitoneally injected with either proteins or PBS at 36 h intervals for 12 weeks, then sacrificed 2 h after the last of these injections. The aortic roots were embedded in OCT (SAKURA, USA) and quickly frozen to −20 °C; then 8-μm serial sections of the aortic root were collected for atherosclerosis analysis. Aortas fixed in 4 % paraformaldehyde were analyzed *en face*. The extent of atherosclerosis was determined by Oil Red O staining and quantification with Image J software as described previously [32].

The location of MsrA in the aortic root was detected by immunofluorescence staining with anti-MsrA antibody. Rat anti-mouse monocytes/macrophage antibody-2 (MOMA2, Bio-rad, USA) was used for macrophage detection in the aortic root. Immunoreactivity was visualized using the Vectastain ABC kit (Vector Labs, USA) and then reacted with DAB substrate (ZSGB-BIO, China). Analysis of apoptosis in aortic root tissue was performed using the terminal deoxynucleotidyl transferase-mediated dUTP nickend-labeling assay kit (TUNEL, Roche Diagnostics, Germany) according to the manufacturer’s instructions. The TUNEL positive cells were counted and normalized to the cell totals. MOMA-2 staining area and apoptosis were analyzed with Image-Pro Plus 6.0.

### Statistical analysis

Data are represented as the mean ± SEM. Statistical analyses were performed using Student’s *t* test and one-way ANOVA between the groups. Differences were considered to be significant at *P* < 0.05.

## Results

### Purified PEP-1-MsrA had similar structure and enzyme activity to MsrA

**Fig. 1:**
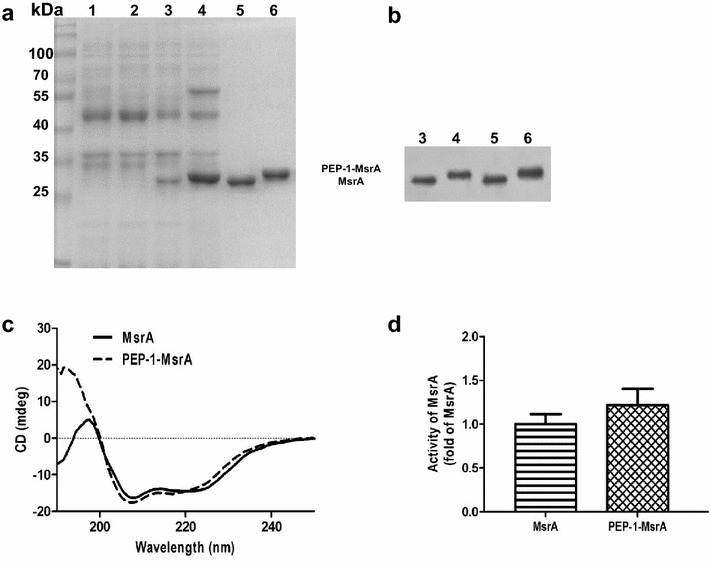
Purification and identification of PEP-1-MsrA and MsrA protein. **a** Expression and purification of the fusion proteins were analyzed by 10 % SDS-PAGE. *1* Non-induced MsrA, *2* non-induced PEP-1-MsrA, *3* induced MsrA, *4* induced PEP-1-MsrA, *5* purified MsrA, *6* purified PEP-1-MsrA. **b** MsrA expression in *E. coli* were detected by Western blot. **c** CD spectra of the pure proteins. **d** MsrA activity was measured using DMSO as the substrate

Recombinant pET28a/MsrA and pET28a/PEP-1-MsrA plasmids were constructed (Additional file 1: Figure S1). PEP-1-MsrA or MsrA proteins were highly expressed in *E. coli* BL21 (DE3) induced by IPTG and were purified from bacteria extracts using Ni-NTA affinity chromatography. The purified proteins were checked by 10 % SDS-PAGE and shown to be more than 95 % purity (Fig. 1a). MsrA proteins were confirmed by Western blot (Fig. 1b). The molecular weights of MsrA and PEP-1-MsrA are ~27 and ~30 kDa, respectively.

To determine whether fusion with PEP-1 could alter the structure of MsrA, we compared the secondary structural features of PEP-1-MsrA and MsrA by far-UV CD. Both PEP-1-MsrA and MsrA adopt a predominantly α-helix conformation with double minima at 208 and 222 nm and have similar α-helix contents (17.1 % for PEP-1-MsrA and 18.1 % for MsrA) (Fig. 1c), suggesting that the fusion with PEP-1 does not change the structure of MsrA.

We also compared the enzyme activity of purified PEP-1-MsrA to that of MsrA. The enzyme activity of PEP-1-MsrA appeared to be slightly higher than that of MsrA, but the difference was not statistically significant (Fig. 1d), indicating that the fusion with PEP-1 does not compromise the function of MsrA.

### Transduction of PEP-1-MsrA into macrophages

**Fig. 2:**
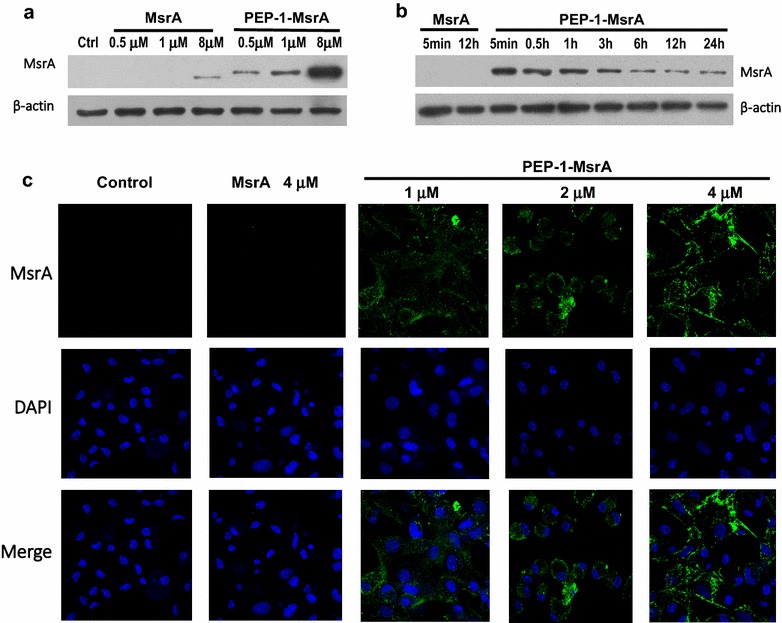
PEP-1-MsrA was transduced into macrophages. Mouse peritoneal macrophages was incubated with PEP-1-MsrA or MsrA (0.5–8 μM) for 1 h (**a**) and 1 μM protein for various times (5 min–24 h) (**b**), then the intracellular MsrA was analyzed by Western blot. **c** The intracellular distribution of PEP-1-MsrA or MsrA in macrophages was determined by confocal microscopy. FITC-conjugated antibody was used for immunofluorescence assay and micrographs were captured at ×1000 magnification

The cytotoxicity of proteins was tested on HeLa cells using the MTT method. We found that even at concentrations up to 18 μM, neither purified MsrA nor PEP-1-MsrA protein reduced cell viability (Additional file 2: Figure S2). We selected 0.5–8 μM purified protein to determine the transduction ability and intracellular stability of PEP-1-MsrA protein. As shown in Fig. 2a, PEP-1-MsrA protein could successfully enter the cells in a dose-dependent manner, whereas control MsrA protein did not enter the cells. Also, peritoneal macrophages were treated with 1 μM PEP-1-MsrA proteins; the presence of PEP-1-MsrA protein was maintained for more than 24 h in the cells (Fig. 2b).

In addition, the intracellular distribution of transduced PEP-1-MsrA was detected by confocal microscopy. As shown in Fig. 2c, PEP-1-MsrA protein was detected in the cytoplasm, and immunofluorescence was increased in a concentration dependent manner, whereas MsrA protein was not detected in macrophages even when incubated with higher concentrations of MsrA protein.

### PEP-1-MsrA inhibited H_2_O_2_ induced oxidation and cell death of macrophages

**Fig. 3:**
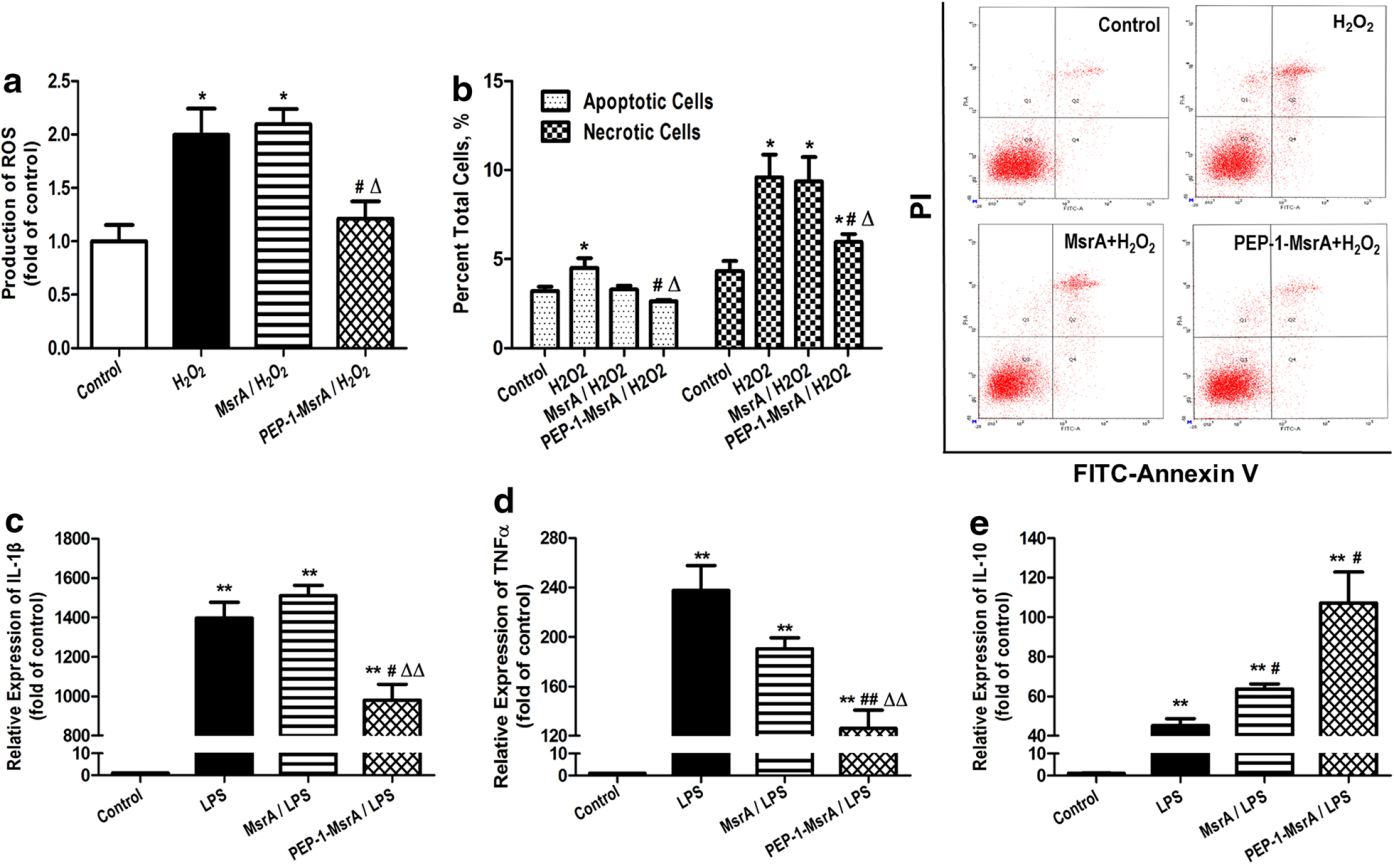
PEP-1-MsrA inhibited oxidation, cell death and inflammation in macrophages. **a** Intracellular ROS levels were measured by flow cytometry. RAW264.7 macrophages were pretreated with PEP-1-MsrA or MsrA (1 µM) and stimulated by 1 mM H_2_O_2_ for 1 h. **b** The apoptosis of mouse peritoneal macrophages induced by H_2_O_2_ was quantified by flow cytometry. **c**–**e** The mRNA levels of inflammation-associated cytokines IL-1β, TNFα and IL-10 were determined by q-PCR. Mouse peritoneal macrophages were treated with 25 ng/ml LPS, and 1 µM PEP-1-MsrA or MsrA for 3 h before mRNA was extracted. n = 3–4 for each group, *P < 0.05, **P < 0.01 vs control group. ^#^ *P* < 0.05, ^##^ *P* < 0.01 vs LPS-induced group. ^Δ^ *P* < 0.05, ^ΔΔ^ *P* < 0.01 vs MsrA-treated group

To determine the effects of transduced PEP-1-MsrA on oxidative stress, RAW264.7 cells were pre-treated with 1 µM PEP-1-MsrA or MsrA proteins for 1 h followed by treatment with 1 mM H_2_O_2_. ROS levels were then measured. As shown in Fig. 3a, H_2_O_2_ treatment resulted in high ROS levels in the cells. ROS production was markedly reduced in the cells pre-treated with PEP-1-MsrA, but was not changed by MsrA pre-treatment. We also examined the impact of PEP-1-MsrA on cell survival under H_2_O_2_ induced oxidative stress using peritoneal macrophages. Compared to MsrA treated cells, cells treated with PEP-1-MsrA showed a remarkable reduction in apoptosis and necrosis (Fig. 3b), indicating that intracellular PEP-1-MsrA could protect macrophages from H_2_O_2_-induced cell death by reducing intracellular ROS levels.

### PEP-1-MsrA suppressed LPS induced inflammation in macrophages

To investigate the effects of intracellular MsrA on inflammatory responses, mouse peritoneal macrophages were pre-treated with PEP-1-MsrA or MsrA and then treated with LPS to induce inflammatory responses. In PEP-1-MsrA pre-treated cells, LPS-induced expression of the proinflammatory cytokines IL-1β and TNFα was significantly inhibited, while the expression of anti-inflammatory cytokine IL-10 was increased compared to the respective levels in MsrA pre-treated cells (Fig. 3c–e), indicating that intracellular PEP-1-MsrA significantly suppressed the macrophage inflammatory response.

### PEP-1-MsrA ameliorated inflammatory and oxidative stress in apoE^−/−^ mice

**Fig. 4:**
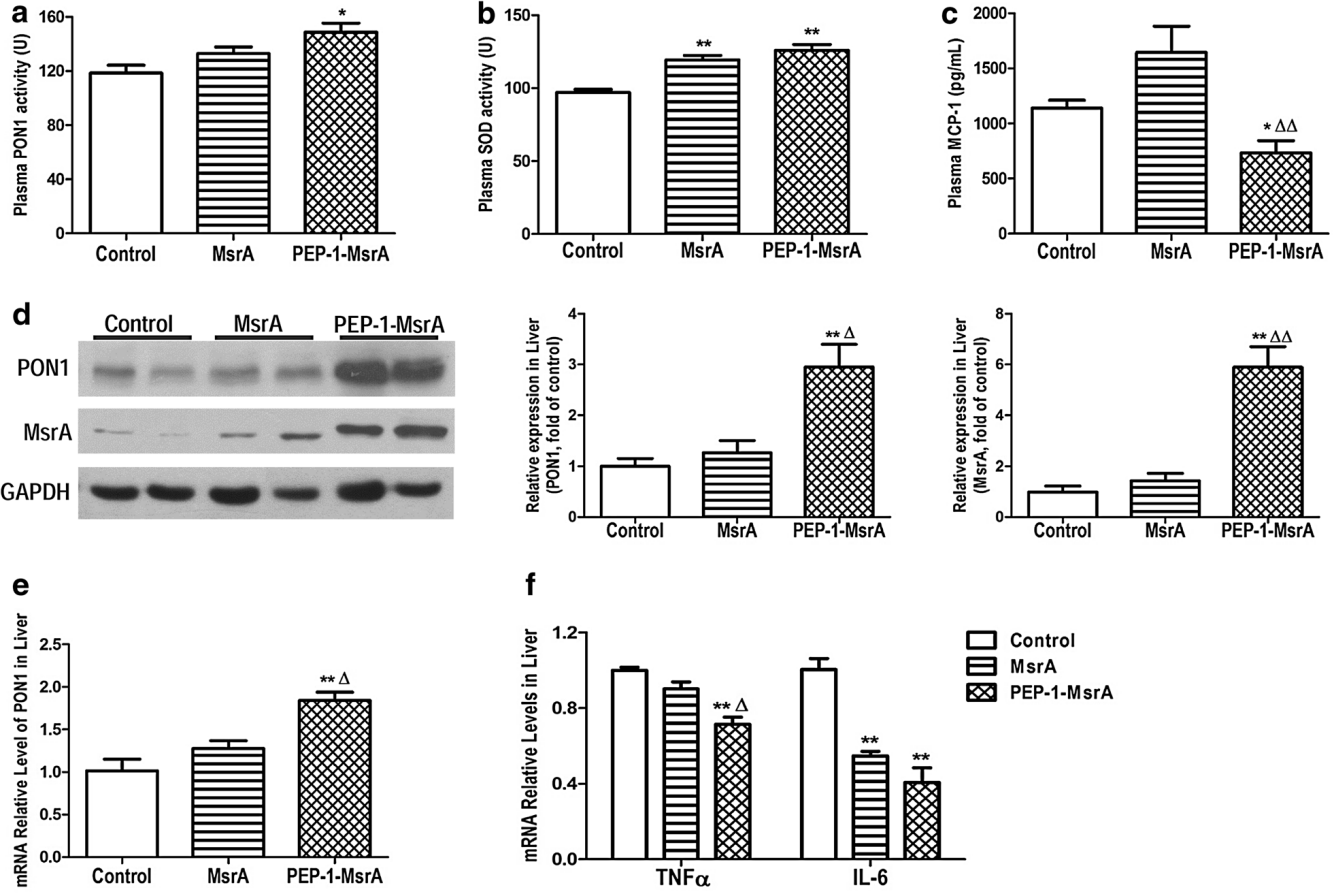
PEP-1-MsrA ameliorated oxidation and inflammation in the plasma and the liver of apoE^−/−^ mice. The 21-week-old apoE^−/−^ mice were fed a Western diet and intraperitoneally injected with PBS, MsrA or PEP-1-MsrA proteins for 12 weeks. **a**, **b** Plasma PON1 and SOD activities were determined, n = 8–10. **c** Plasma MCP-1 level was detected by ELISA, n = 10. **d** MsrA and PON1 levels in the liver were determined by Western blot. **e** PON1 mRNA levels in mice liver were measured by qPCR. **f** The mRNA levels of TNFα and IL-6 in mice liver were measured by qPCR. All tests were analyses of three independent experiments (n = 4–5). *P < 0.05, **P < 0.01 vs control group, ^Δ^ *P* < 0.05, ^ΔΔ^ *P* < 0.01 vs MsrA group

ApoE^−/−^ mice were intraperitoneally injected with MsrA, PEP-1-MsrA proteins or vehicle and fed a Western-diet for 12 weeks. The body weights (at various time-points) and spleen weights (at the end point) of mice showed no difference between all three groups (Additional file 3: Figure S3). Plasma PON1 and SOD activities are the indicators of anti-oxidative status in circulation. We found that plasma PON1 and SOD activities were significantly increased, while the plasma inflammatory factor MCP-1 level was decreased in PEP-1-MsrA injected mice compared to the respective levels in control mice or MsrA injected mice (Fig. 4a–c).

The liver is an important organ for the regulation of oxidative and inflammatory status. PON1 is an HDL-associated antioxidase that is mainly synthesized in liver. We found that mRNA and protein levels of PON1 in liver tissue were significantly increased in PEP-1-MsrA injected mice compared to those of MsrA injected or control mice. This is consistent with the high level of MsrA in the livers of PEP-1-MsrA injected mice (Fig. 4d, e). Meanwhile, the mRNA levels of inflammatory cytokines TNFα and IL-6 were significantly decreased in PEP-1-MsrA treated mice (Fig. 4f). These data indicate that when PEP-1-MsrA enters the cells in the liver, it plays an anti-oxidative and anti-inflammatory role through upregulating PON1 expression and inhibiting the expression of inflammatory cytokines. These data demonstrated that transduced PEP-1-MsrA improved circulation and hepatic anti-oxidative and anti-inflammatory status in apoE^−/−^ mice.

### PEP-1-MsrA attenuated atherosclerosis in apoE^−/−^ mice

**Fig. 5:**
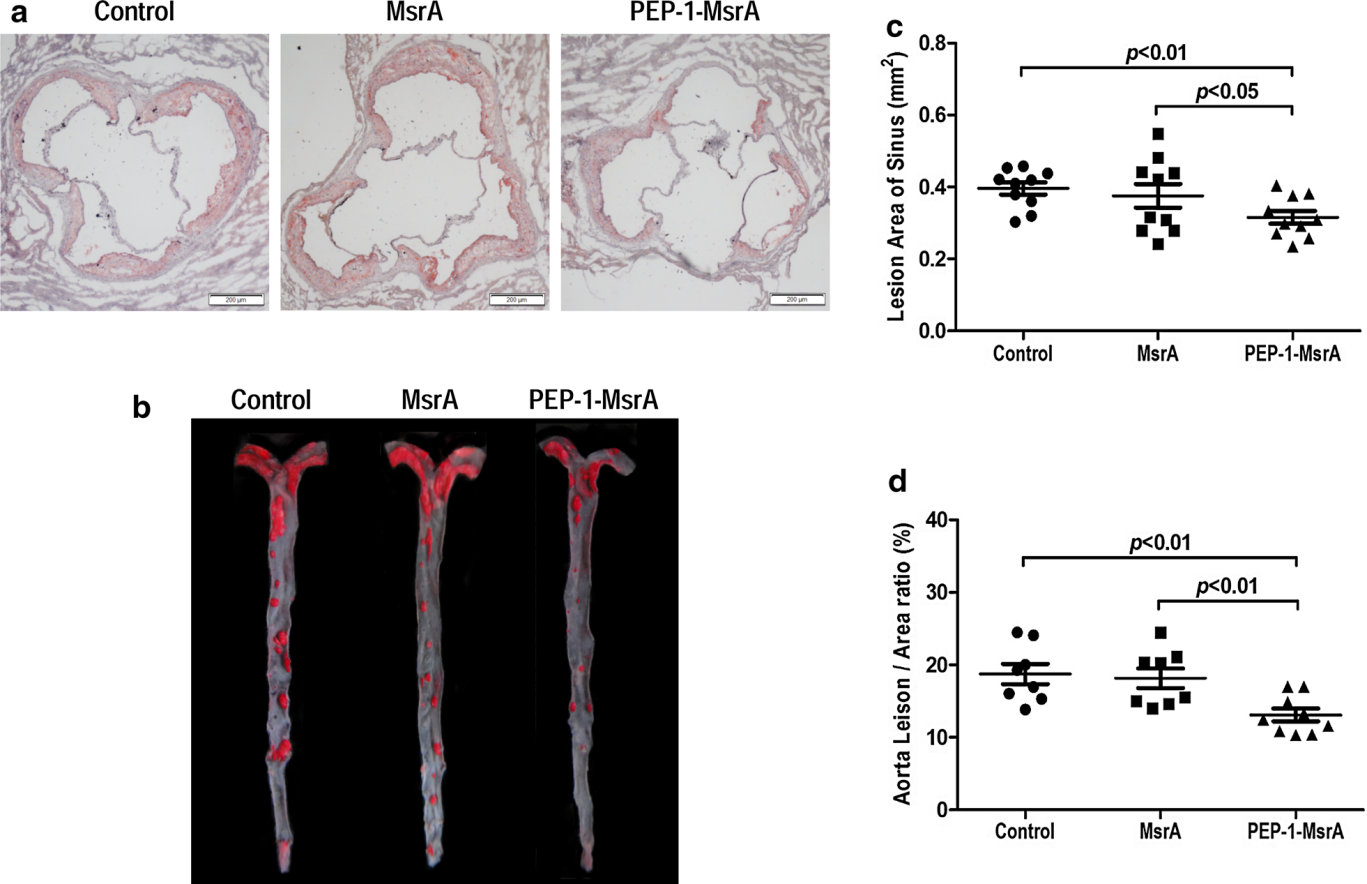
PEP-1-MsrA reduced atherosclerotic lesions in apoE^−/−^ mice. The atherosclerotic lesions was examined using Oil Red O-stained cross-sections of the aorta root (8-µm serial sections) and by *en face* analysis of the aorta. **a**, **b** Representative lesions in cross-sections of the aorta root and *en face* aortas. **c**, **d** The atherosclerotic lesion areas were quantified with Image J software

During the treatment with purified proteins and Western-diet feeding, the plasma TC and TG levels were not significantly different between the three groups (Table 1). The impact of PEP-1-MsrA injection on the development of atherosclerosis in apoE^−/−^ mice was assessed. Representative atherosclerotic lesions in cross-sections of aortic roots and *en face* aorta images stained with Oil Red O are shown in Fig. 5a, b. The lipid staining area in the lesion of PEP-1-MsrA injected mice (0.32 ± 0.02 mm^2^) was significantly smaller than that in control mice (0.40 ± 0.02 mm^2^) or MsrA injected mice (0.38 ± 0.03 mm^2^), reduced by 20 and 16 % respectively (*P* < 0.01 or *P* < 0.05, Fig. 5c). *En face* analysis of pinned-out aortas revealed that the percent area of atherosclerotic lesion in PEP-1-MsrA injected mice was also significantly reduced compared to MsrA injected mice and control mice (*P* < 0.01, Fig. 5d).

**Table 1:** Plasma lipids levels in apoE^−/−^ mice

	Time (weeks)	Control	MsrA	PEP-1-MsrA
TC (mg/dL)	0	522.9 ± 89.1	561.6 ± 76.6	528.0 ± 28.1
	4	696.7 ± 116.9	727.1 ± 92.8	699.7 ± 83.7
	8	824.6 ± 67.5	809.1 ± 126.9	863.8 ± 143.1
	12	888.6 ± 75.7	929.9 ± 91.7	867.3 ± 119.1
TG (mg/dL)	0	127.4 ± 19.7	139.3 ± 25.4	137.7 ± 21.3
	4	170.4 ± 20.7	164.9 ± 22.0	168.3 ± 16.1
	8	188.0 ± 30.7	206.2 ± 40.4	192.6 ± 35.7
	12	191.5 ± 41.5	185.3 ± 30.0	194.3 ± 30.6

Data are given as the mean ± SEM, n = 10. The statistical analysis was performed using a two-tailed Student’s *t* test. Mice were fed a Western-type diet and intraperitoneally injected with PBS, MsrA or PEP-1-MsrA proteins for 12 weeks

*TC* total cholesterol, *TG* triglycerides

**Fig. 6:**
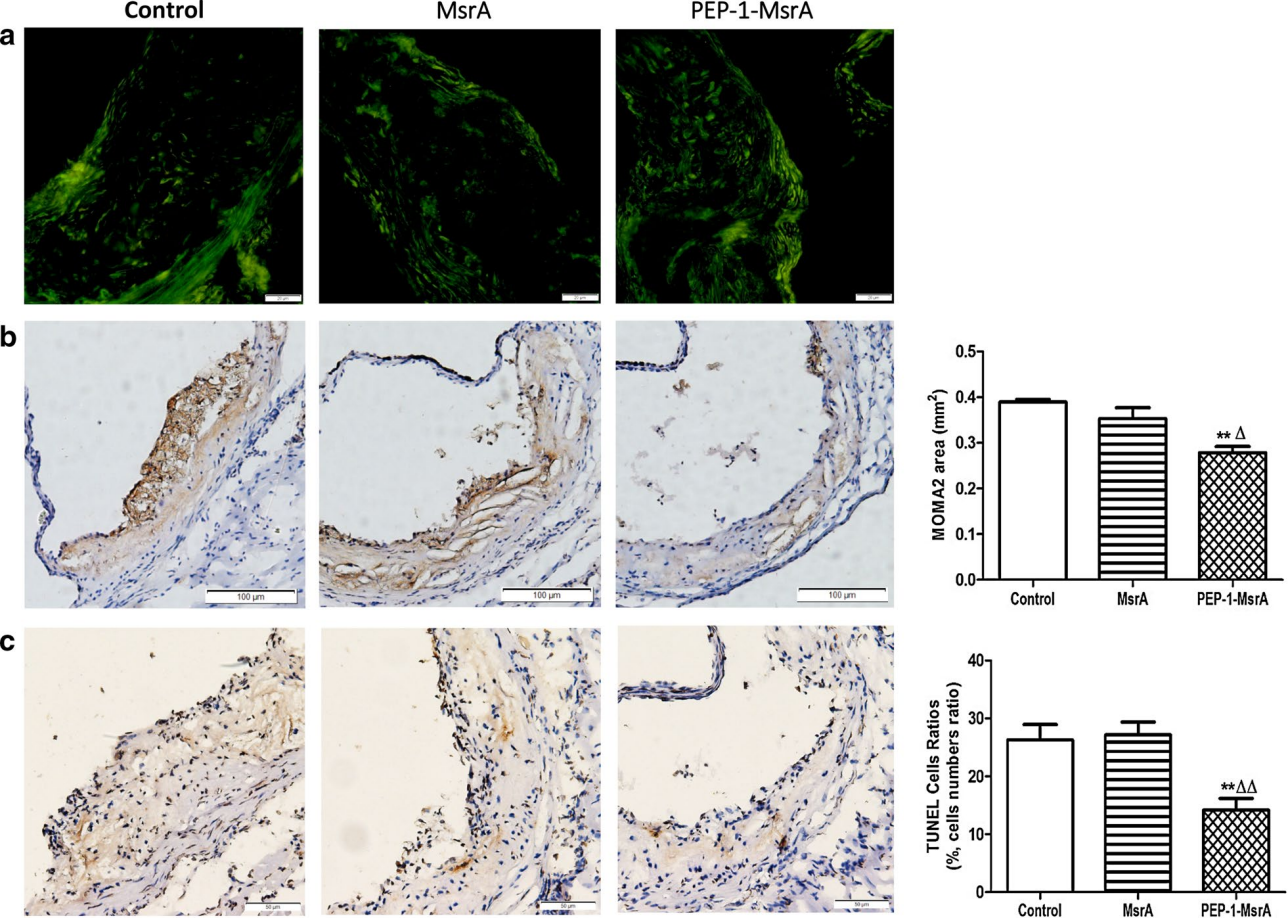
PEP-1-MsrA reduced macrophage infiltration and cell apoptosis in the lesions. **a** The MsrA level in the aortic lesions was detected by immunofluorescence assay. **b** The macrophages in aortic root lesions were visualized by Immunostaining for MOMA-2 and MOMA-2 staining area was analyzed with Image-Pro Plus 6.0. **c** Apoptosis in lesions was detected by TUNEL assay. The apoptotic cells were counted and normalized to the total number of cells in the lesion area

We found that higher levels of MsrA were detected in the arterial walls of PEP-1-MsrA injected mice compared to those in the control and MsrA groups, indicating that PEP-1-MsrA successfully entered the cells of aorta (Fig. 6a). We further stained macrophages in aortic root lesions using a mouse macrophage specific antibody, MOMA2, and found the PEP-1-MsrA injected mice displayed 28 % smaller macrophage areas compared to control mice (0.28 ± 0.01 vs 0.39 ± 0.01 mm^2^) and 20 % smaller macrophage areas compared with MsrA injected mice (0.35 ± 0.02 mm^2^) (Fig. 6b). In addition, there were 46 % fewer apoptotic cells in the aortic root cross-sections of PEP-1-MsrA injected mice than in those of control mice and 48 % fewer than in MsrA injected mice (Fig. 6c). These data show that PEP-1-MsrA attenuates the development of atherosclerosis in apoE^−/−^ mice by reducing the accumulation of macrophages, especially apoptotic macrophages, in the lesions.

## Discussion

Intracellular redox status is tightly regulated by oxidant and antioxidant systems. An imbalance between these systems causes ROS accumulation which leads to oxidative stress and inflammation. It has been shown that ROS-induced oxidation is a well-known cause of atherogenesis and is involved in every step of atherosclerosis development, including vascular endothelial cell injury, macrophage chemotaxis, foam cell formation and smooth muscle cell migration and proliferation [33]. Many research efforts have been made without much success to develop a therapeutic approach for atherosclerosis through regulating the redox status by improving the efficacy of antioxidant enzymes.

MsrA, one of the antioxidant defenses in cells, is important in the maintenance of redox homeostasis and in the prevention of oxidative stress-related disease. MsrA is distributed in the cytoplasm, mitochondria and nuclei and is involved in regulating the redox state and the function of numerous proteins, for examples, calmodulin, apolipoprotein AI, IL-6, interferon and cytochrome c oxidase [34]. Studies have shown that MsrA activity was significantly reduced in the brains of Alzheimer’s disease patients and in the cardiomyocytes in cardiac ischemia models [35, 36]. *E. coli* and yeast with MsrA mutations or deficiency were particularly sensitive to oxidative damage [37, 38]. MsrA knockout mice are highly sensitive to oxidative stress and show nerve damage and shortened lifespans [39]. Whereas MsrA transgenic *Drosophila* show significantly enhanced anti-oxidation and anti-aging characteristics [40]. However, whether or not MsrA could be used as a therapeutic agent for atherosclerosis has not been investigated.

Due to their size and low permeability, it is usually difficult for foreign proteins to enter cells and perform intracellular functions [18]. A recent report showed that the 21-residue PEP-1 peptide is a new type of amphipathic CPP [20]. Several PEP-1 fusion proteins, such as PEP-1-SOD, PEP-1-CAT, PEP-1-SIRT2 and PEP-1-PON1, have been designed; and studies have demonstrated that PEP-1 is a promising tool that may help in the development of potential protein therapeutic agents against oxidative stress [21–23, 28]. We constructed a PEP-1-MsrA fusion protein in order to deliver MsrA into cells and allow it to regenerate its reductase activity using the intrinsic cellular Trx reduction system. We found that MsrA, fused with a PEP-1 peptide, was able to efficiently enter macrophages with regular incubation. Lee et al. recently reported that a similar fusion protein of MsrA could rapidly enter keratinocytes and play a protective role against oxidative stress and ultraviolet radiation-induced cell death [41]. Interesting, they found that their fusion protein was truncated in the N-terminal region of MsrA between Lys-27 and Val-28 during expression in *E. coli* and purification. MsrA in our study was designed to cleave off N-terminal domain of mitochondrial targeting signal peptide, so we did not observe the phenomenon of MsrA protein truncation in vitro. Our study showed that PEP-1-MsrA entered into macrophages and exerted the anti-oxidative and anti-inflammatory functions through decreasing intracellular ROS levels. Also of note, PEP-1-MsrA suppressed LPS-induced expression of the proinflammatory cytokines TNFα and IL-1β and increased expression of the anti-inflammatory cytokine IL-10 [42], suggesting that intracellular MsrA could inhibit M1 polarization of macrophages under pro-inflammatory stimulations.

In the in vivo study, we injected purified PEP-1-MsrA proteins into apoE^−/−^ mice fed a Western diet. ApoE^−/−^ mice have been widely used as an atherosclerotic mice model due to the development of spontaneous hypercholesterolemia and atherosclerosis, which can be accelerated by high fat diet feeding. We found that injection of PEP-1-MsrA could significantly reduce the size of atherosclerotic lesions without altering plasma lipid levels, but injection of MsrA did not affect atherosclerosis. These results suggest that the anti-oxidative and anti-inflammatory effects of cell penetrating PEP-1-MsrA confer anti-atherogenic benefit to the hyperlipidemic mice through lipid-independent mechanisms. In fact, our results showed that PEP-1-MsrA injection increased mouse plasma PON1 and SOD activities and decreased plasma MCP-1 levels. PON1 is mainly synthesized in the liver and secreted to the plasma where it is mainly associated with HDL particles. Our previous study indicated the negative correlation between the activity of plasma PON1 and the extent of atherosclerosis [31]. SOD is another important protein that maintains redox homeostasis. A recent study reported that Met/MetSO modified liver homeostasis and altered the redox state by increasing the activity of SOD [43]. Our results further demonstrated that accumulation of PEP-1-MsrA in the liver led to an increase in hepatic PON1 mRNA and protein levels, indicating that PEP-1-MsrA may enter hepatocytes and up-regulate PON1 expression. In the meantime, the expression of inflammatory cytokines TNFα and IL-6 was also reduced in the liver of the PEP-1-MsrA injected mice. Interestingly, the injection of MsrA, although largely ineffective, indeed also increased plasma SOD activity and reduced hepatic IL-6 expression. These data indicate the extracellular MsrA may exert mild anti-oxidative and anti-inflammatory function in the liver, but not robust enough to reduce atherosclerosis.

Another important mechanism by which PEP-1-MsrA reduces atherosclerosis, however, may lie in its effects on macrophages. First, our in vitro data clearly showed that PEP-1-MsrA could effectively enter macrophages and exert anti-oxidative and anti-inflammatory function as mentioned earlier. Second, PEP-1-MsrA injection reduced plasma MCP-1 levels. Although we do not know in what organs and cell types MCP-1 production was inhibited by PEP-1-MsrA, the reduced plasma MCP-1 levels would lead to reduced monocyte/macrophage infiltration to the arterial wall [44]. Third, in fact, macrophage content in the lesions of PEP-1-MsrA injected mice was significantly reduced. Fourth, more interestingly, the apoptotic macrophages were even more reduced in lesions of PEP-1-MsrA injected mice than total macrophages, suggesting the apoptosis-preventive function of PEP-1-MsrA shown in vitro also operated in the atherosclerotic lesions; and actually PEP-1-MsrA protein was detected enriched in the lesions.

## Conclusions

MsrA, as a specific intracellular MetSO reductase, plays an important role in redox homeostasis. Our studies indicate that PEP-1-MsrA effectively enter cells and plays a protective role against oxidative stress and inflammation. In Western diet-fed apoE^−/−^ mice model, administration of this protein could significantly halt atherogenesis through increasing the anti-oxidative and anti-inflammatory capacity in the liver and reducing macrophage accumulation, inflammatory responses, and apoptosis in the lesions. Therefore, PEP-1-MsrA may be developed as a potent therapeutic agent for reducing atherosclerosis-related cardiovascular diseases.

## Additional files

10.1186/s12967-015-0677-8 Vector diagrams of pET28a/MsrA and pET28a/PEP-1-MsrA. A 642-bp cDNA fragment of human MsrA was inserted into pET28a vector, PEP-1 was inserted at the N-terminus of MsrA.
10.1186/s12967-015-0677-8 Cell viability analysis of MsrA and PEP-1-MsrA on HeLa cells. Different concentrations of MsrA and PEP-1-MsrA proteins were incubated with HeLa cells for 72 h. Cell viability was analyzed by MTT method, n = 3.
10.1186/s12967-015-0677-8 Effects of MsrA or PEP-1-MsrA on body weight, spleen weight of apoE^−/−^ mice. The 21-week-old apoE^−/−^ mice were fed with a Western-type diet and intraperitoneally injected with PBS, MsrA or PEP-1-MsrA proteins for 12 weeks and then sacrificed. The body weights at various time-points (**A**) and spleen weights at the end point (**B**) were measured, n = 10.

## Abbreviations

CAT: catalase
CD: circular dichroism
CPPs: cell penetrating peptides
CVD: cardiovascular disease
IL: interleukin
LPS: lipopolysaccharide
MCP-1: monocyte chemoattractant protein 1
MetSO: methionine-*S*-sulfoxide
MsrA: methionine sulfoxide reductase A
PBS: phosphate-buffered saline
PON1: paraoxonase-1
PTDs: protein transduction domains
ROS: reactive oxygen species
SOD: superoxide dismutase
TC: total cholesterol
TG: triglycerides
Trx: thioredoxin

## References

[1] Hopkins PN (2013). Molecular biology of atherosclerosis. Physiol Rev.

[2] Liu SX, Hou FF, Guo ZJ, Nagai R, Zhang WR, Liu ZQ (2006). Advanced oxidation protein products accelerate atherosclerosis through promoting oxidative stress and inflammation. Arterioscler Thromb Vasc Biol.

[3] Libby P, Lichtman AH, Hansson GK (2013). Immune effector mechanisms implicated in atherosclerosis: from mice to humans. Immunity.

[4] Briley-Saebo KC, Cho YS, Tsimikas S (2011). Imaging of oxidation-specific epitopes in atherosclerosis and macrophage-rich vulnerable plaques. Curr Cardiovasc Imaging Rep..

[5] Madamanchi NR, Runge MS (2013). Redox signaling in cardiovascular health and disease. Free Radic Biol Med.

[6] Madamanchi NR, Vendrov A, Runge MS (2005). Oxidative stress and vascular disease. Arterioscler Thromb Vasc Biol.

[7] Li H, Horke S, Forstermann U (2013). Oxidative stress in vascular disease and its pharmacological prevention. Trends Pharmacol Sci.

[8] Kim HY (2013). The methionine sulfoxide reduction system: selenium utilization and methionine sulfoxide reductase enzymes and their functions. Antioxid Redox Signal.

[9] Moskovitz J (2005). Roles of methionine sulfoxide reductases in antioxidant defense, protein regulation and survival. Curr Pharm Des.

[10] Prentice HM, Moench IA, Rickaway ZT, Dougherty CJ, Webster KA, Weissbach H (2008). MsrA protects cardiac myocytes against hypoxia/reoxygenation induced cell death. Biochem Biophys Res Commun..

[11] Kim JI, Choi SH, Jung KJ, Lee E, Kim HY, Park KM (2013). Protective role of methionine sulfoxide reductase A against ischemia/reperfusion injury in mouse kidney and its involvement in the regulation of trans-sulfuration pathway. Antioxid Redox Signal.

[12] Brennan LA, Kantorow M (2009). Mitochondrial function and redox control in the aging eye: role of MsrA and other repair systems in cataract and macular degenerations. Exp Eye Res.

[13] Styskal J, Nwagwu FA, Watkins YN, Liang H, Richardson A, Musi N (2013). Methionine sulfoxide reductase A affects insulin resistance by protecting insulin receptor function. Free Radic Biol Med.

[14] Schoneich C (2005). Methionine oxidation by reactive oxygen species. Reaction mechanisms and relevance to Alzheimer’s disease. Biochim Biophys Acta.

[15] Misiti F, Clementi ME, Giardina B (2010). Oxidation of methionine 35 reduces toxicity of the amyloid beta-peptide(1–42) in neuroblastoma cells (IMR-32) via enzyme methionine sulfoxide reductase A expression and function. Neurochem Int.

[16] Garcia-Bermudez M, Lopez-Mejias R, Gonzalez-Juanatey C, Castaneda S, Miranda-Filloy JA, Blanco R (2012). Association of the methionine sulfoxide reductase A rs10903323 gene polymorphism with cardiovascular disease in patients with rheumatoid arthritis. Scand J Rheumatol.

[17] Gu H, Chen W, Yin J, Chen S, Zhang J, Gong J (2013). Methionine sulfoxide reductase A rs10903323 G/A polymorphism is associated with increased risk of coronary artery disease in a Chinese population. Clin Biochem.

[18] Deshayes S, Morris M, Heitz F, Divita G (2008). Delivery of proteins and nucleic acids using a non-covalent peptide-based strategy. Adv Drug Deliv Rev.

[19] van den Berg A, Dowdy SF (2011). Protein transduction domain delivery of therapeutic macromolecules. Curr Opin Biotechnol.

[20] Morris MC, Depollier J, Mery J, Heitz F, Divita G (2001). A peptide carrier for the delivery of biologically active proteins into mammalian cells. Nat Biotechnol.

[21] Huang GQ, Wang JN, Tang JM, Zhang L, Zheng F, Yang JY (2011). The combined transduction of copper, zinc-superoxide dismutase and catalase mediated by cell-penetrating peptide, PEP-1, to protect myocardium from ischemia-reperfusion injury. J Transl Med..

[22] Zhang L, Wei S, Tang JM, Guo LY, Zheng F, Yang JY (2013). PEP-1-CAT protects hypoxia/reoxygenation-induced cardiomyocyte apoptosis through multiple signaling pathways. J Transl Med..

[23] Kim MJ, Jeong HJ, Kim DW, Sohn EJ, Jo HS, Kim DS (2014). PEP-1-PON1 protein regulates inflammatory response in raw 264.7 macrophages and ameliorates inflammation in a TPA-induced animal model. PLoS One.

[24] Jeong HJ, Yoo DY, Kim DW, Yeo HJ, Cho SB, Hyeon J (2014). Neuroprotective effect of PEP-1-peroxiredoxin2 on CA1 regions in the hippocampus against ischemic insult. Biochim Biophys Acta.

[25] Kurzawa L, Pellerano M, Morris MC (2010). PEP and CADY-mediated delivery of fluorescent peptides and proteins into living cells. Biochim Biophys Acta.

[26] Kim DW, Kim DS, Kim MJ, Kwon SW, Ahn EH, Jeong HJ (2011). Imipramine enhances neuroprotective effect of PEP-1-catalase against ischemic neuronal damage. BMB Rep..

[27] Zhang YE, Wang JN, Tang JM, Guo LY, Yang JY, Huang YZ (2009). In vivo protein transduction: delivery of PEP-1-SOD1 fusion protein into myocardium efficiently protects against ischemic insult. Mol Cells.

[28] Kim MJ, Kim DW, Park JH, Kim SJ, Lee CH, Yong JI (2013). PEP-1-SIRT2 inhibits inflammatory response and oxidative stress-induced cell death via expression of antioxidant enzymes in murine macrophages. Free Radic Biol Med.

[29] Wu PF, Zhang Z, Guan XL, Li YL, Zeng JH, Zhang JJ (2013). A specific and rapid colorimetric method to monitor the activity of methionine sulfoxide reductase A. Enzym Microb Technol.

[30] Du F, Yu F, Wang Y, Hui Y, Carnevale K, Fu M (2014). MicroRNA-155 deficiency results in decreased macrophage inflammation and attenuated atherogenesis in apolipoprotein E-deficient mice. Arterioscler Thromb Vasc Biol.

[31] Zhou C, Cao J, Shang L, Tong C, Hu H, Wang H (2013). Reduced paraoxonase 1 activity as a marker for severe coronary artery disease. Dis Markers.

[32] Yu H, Zhang W, Yancey PG, Koury MJ, Zhang Y, Fazio S (2006). Macrophage apolipoprotein E reduces atherosclerosis and prevents premature death in apolipoprotein E and scavenger receptor-class BI double-knockout mice. Arterioscler Thromb Vasc Biol.

[33] Stocker R, Keaney JFJ (2003). Role of oxidative modification in atherosclerosis. Physiol Rev.

[34] Oien DB, Moskovitz J (2008). Substrates of the methionine sulfoxide reductase system and their physiological relevance. Curr Top Dev Biol.

[35] Gabbita SP, Aksenov MY, Lovell MA, Markesbery WR (1999). Decrease in peptide methionine sulfoxide reductase in Alzheimer’s disease brain. J Neurochem.

[36] Picot CR, Perichon M, Lundberg KC, Friguet B, Szweda LI, Petropoulos I (2006). Alterations in mitochondrial and cytosolic methionine sulfoxide reductase activity during cardiac ischemia and reperfusion. Exp Gerontol.

[37] Moskovitz J, Rahman MA, Strassman J, Yancey SO, Kushner SR, Brot N (1995). Escherichia coli peptide methionine sulfoxide reductase gene: regulation of expression and role in protecting against oxidative damage. J Bacteriol.

[38] Oien D, Moskovitz J (2007). Protein-carbonyl accumulation in the non-replicative senescence of the methionine sulfoxide reductase A (msrA) knockout yeast strain. Amino Acids.

[39] Moskovitz J, Bar-Noy S, Williams WM, Requena J, Berlett BS, Stadtman ER (2001). Methionine sulfoxide reductase (MsrA) is a regulator of antioxidant defense and lifespan in mammals. Proc Natl Acad Sci USA.

[40] Chung H, Kim A-K, Jung S-A, Kim SW, Yu K, Lee JH (2010). The *Drosophila* homolog of methionine sulfoxide reductase A extends lifespan and increases nuclear localization of FOXO. FEBS Lett.

[41] Lee TH, Choi SH, Kim HY (2011). The protein truncation caused by fusion of PEP-1 peptide and protective roles of transduced PEP-1-MsrA in skin cells. BMB Rep..

[42] Von Der Thusen JH, Kuiper J, Fekkes ML, De Vos P, Van Berkel TJ, Biessen EA (2001). Attenuation of atherogenesis by systemic and local adenovirus-mediated gene transfer of interleukin-10 in LDLr^−/−^ mice. FASEB J..

[43] Costa MZ, da Silva TM, Flores NP, Schmitz F, da Silva Scherer EB, Viau CM (2013). Methionine and methionine sulfoxide alter parameters of oxidative stress in the liver of young rats: in vitro and in vivo studies. Mol Cell Biochem.

[44] Lin J, Kakkar V, Lu X (2014). Impact of MCP-1 in atherosclerosis. Curr Pharm Des.

