# Supplementary material for: PEP-1-MsrA ameliorates inflammation and reduces atherosclerosis in apolipoprotein E deficient mice
Source: J Transl Med. 2015 Sep 26;13:316. doi: 10.1186/s12967-015-0677-8 (PMC4584131; doi:10.1186/s12967-015-0677-8)
Supplement: Supplementary file 1 — 10.1186/s12967-015-0677-8 Vector diagrams of pET28a/MsrA and pET28a/PEP-1-MsrA. A 642-bp cDNA fragment of human MsrA was inserted into pET28a vector, PEP-1 was inserted at the N-terminus of MsrA. [file 12967_2015_677_MOESM1_ESM.ppt]

## Slide 1
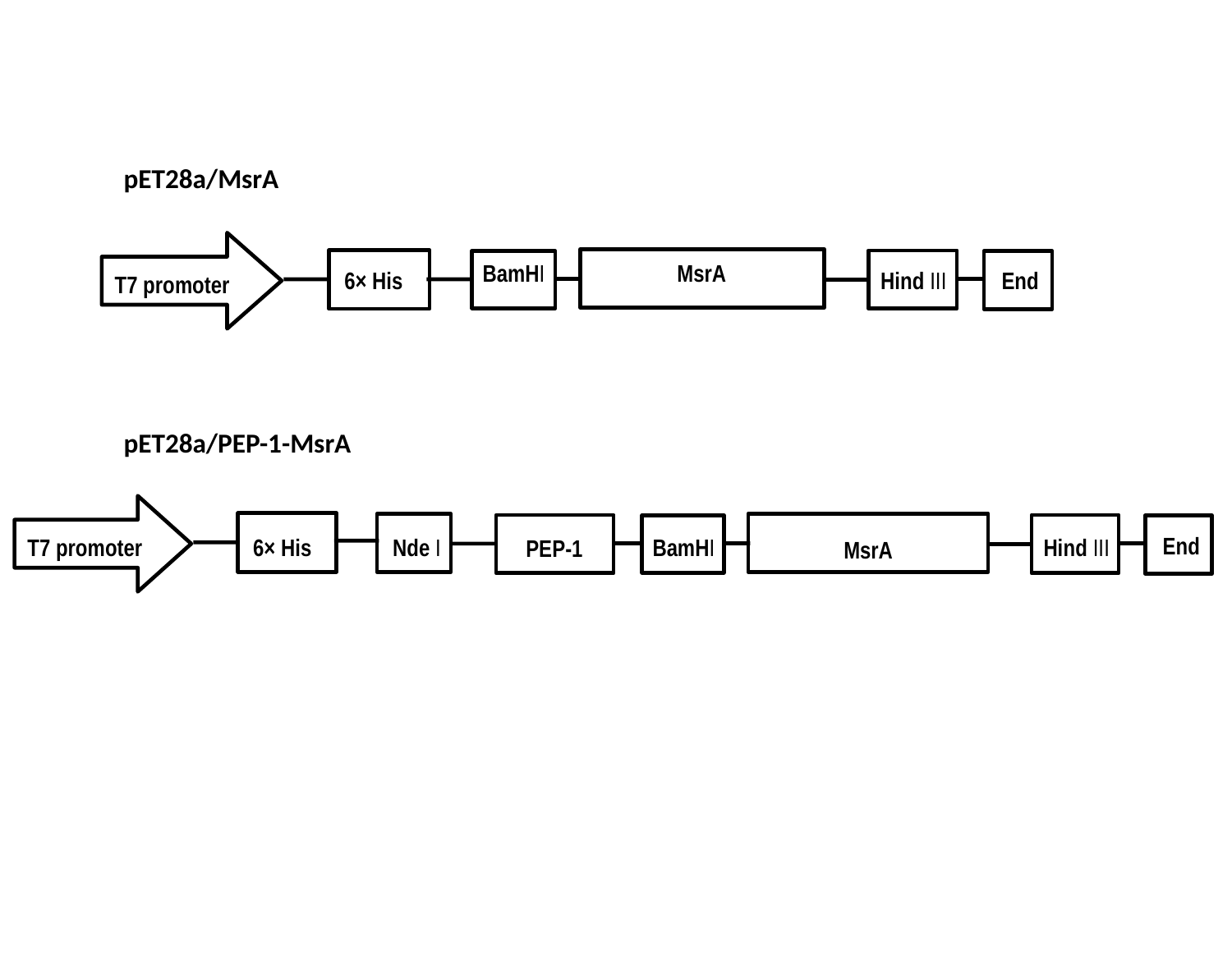

pET28a/MsrA
BamHІ
MsrA
6× His
Hind III
End
T7 promoter
pET28a/PEP-1-MsrA
End
Nde І
Hind III
T7 promoter
6× His
BamHІ
PEP-1
MsrA
