# Supplementary figures and images for: PEP-1-MsrA ameliorates inflammation and reduces atherosclerosis in apolipoprotein E deficient mice
Source: J Transl Med. 2015 Sep 26;13:316. doi: 10.1186/s12967-015-0677-8 (PMC4584131; doi:10.1186/s12967-015-0677-8)

## Slide 1
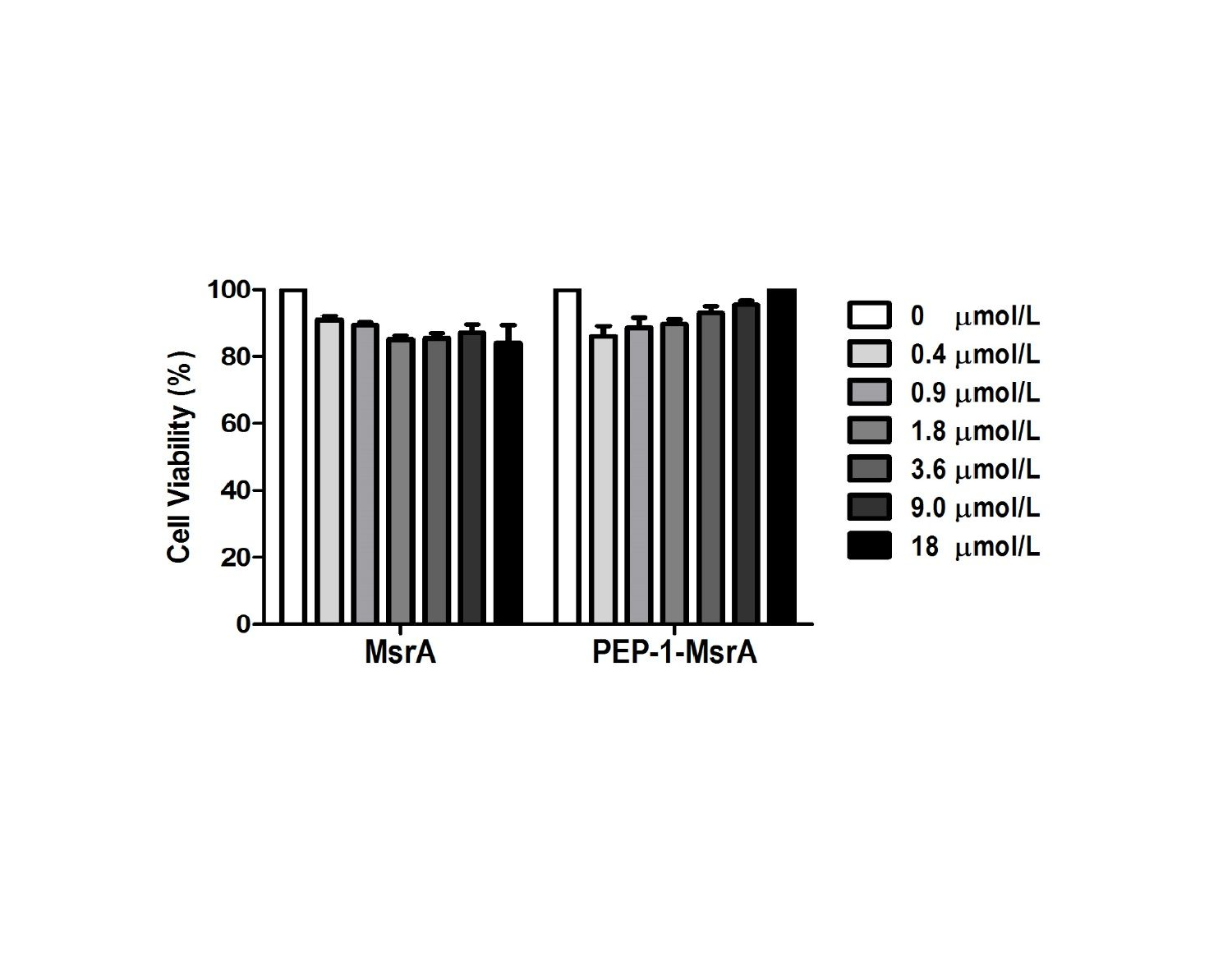

Supplement: Supplementary file 2 — 10.1186/s12967-015-0677-8 Cell viability analysis of MsrA and PEP-1-MsrA on HeLa cells. Different concentrations of MsrA and PEP-1-MsrA proteins were incubated with HeLa cells for 72 h. Cell viability was analyzed by MTT method, n = 3. [file 12967_2015_677_MOESM2_ESM.ppt]

## Slide 1
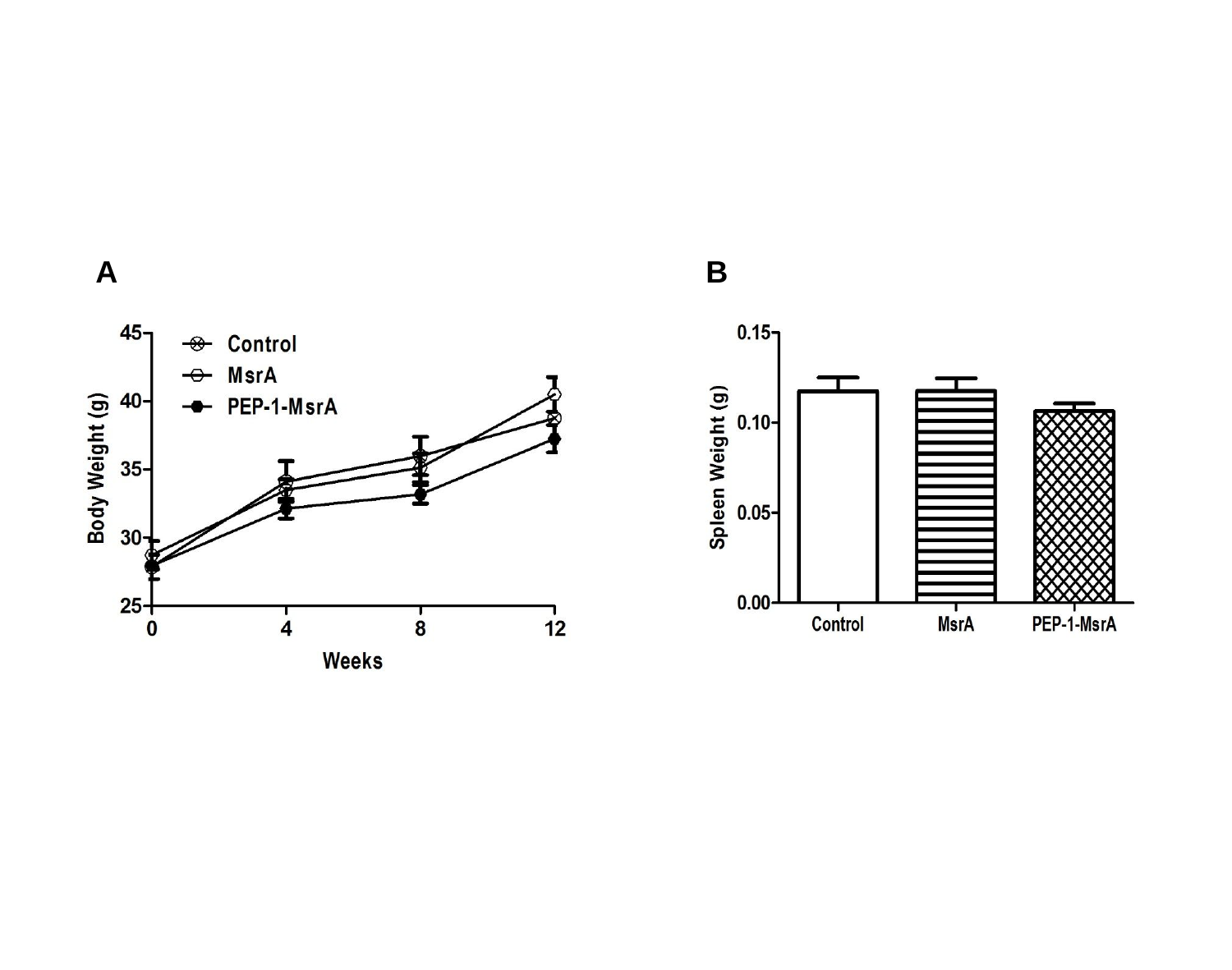

A
B

Supplement: Supplementary file 3 — 10.1186/s12967-015-0677-8 Effects of MsrA or PEP-1-MsrA on body weight, spleen weight of apoE−/− mice. The 21-week-old apoE−/− mice were fed with a Western-type diet and intraperitoneally injected with PBS, MsrA or PEP-1-MsrA proteins for 12 weeks and then sacrificed. The body weights at various time-points (A) and spleen weights at the end point (B) were measured, n = 10. [file 12967_2015_677_MOESM3_ESM.ppt]
